# Rgs13 Constrains Early B Cell Responses and Limits Germinal Center Sizes

**DOI:** 10.1371/journal.pone.0060139

**Published:** 2013-03-22

**Authors:** Il-Young Hwang, Kyung-Sun Hwang, Chung Park, Kathleen A. Harrison, John H. Kehrl

**Affiliations:** B Cell Molecular Immunology Section, Laboratory of Immunoregulation, National Institute of Allergy and Infectious Diseases, National Institutes of Health, Bethesda, Maryland, United States of America; McGill University, Canada

## Abstract

Germinal centers (GCs) are microanatomic structures that develop in secondary lymphoid organs in response to antigenic stimulation. Within GCs B cells clonally expand and their immunoglobulin genes undergo class switch recombination and somatic hypermutation. Transcriptional profiling has identified a number of genes that are prominently expressed in GC B cells. Among them is R*gs13*, which encodes an RGS protein with a dual function. Its canonical function is to accelerate the intrinsic GTPase activity of heterotrimeric G-protein α subunits at the plasma membrane, thereby limiting heterotrimeric G-protein signaling. A unique, non-canonical function of RGS13 occurs following translocation to the nucleus, where it represses CREB transcriptional activity. The functional role of RGS13 in GC B cells is unknown. To create a surrogate marker for *Rgs13* expression and a loss of function mutation, we inserted a GFP coding region into the *Rgs13* genomic locus. Following immunization GFP expression rapidly increased in activated B cells, persisted in GC B cells, but declined in newly generated memory B and plasma cells. Intravital microscopy of the inguinal lymph node (LN) of immunized mice revealed the rapid appearance of GFP^+^ cells at LN interfollicular regions and along the T/B cell borders, and eventually within GCs. Analysis of WT, knock-in, and mixed chimeric mice indicated that RGS13 constrains extra-follicular plasma cell generation, GC size, and GC B cell numbers. Analysis of select cell cycle and GC specific genes disclosed an aberrant gene expression profile in the *Rgs13* deficient GC B cells. These results indicate that RGS13, likely acting at cell membranes and in nuclei, helps coordinate key decision points during the expansion and differentiation of naive B cells.

## Introduction

During a T cell dependent antibody response the engagement of the B cell antigen receptor by cognate antigen initiates an activation program that prepares naïve B cells to receive T cell help [1] One consequence is an increase in their sensitivity to CCR7 and EBI2 ligands, which helps localize the recently antigen activated B cells to the T-B cell border and interfollicular zones, the sites where they receive T cell help and undergo an initial proliferative expansion [2], [3], [4]. These expanding B cells have three fates: an early plasmablast, which is responsible for the initial extra-follicular antibody response; an early memory B cell; or a GC precursor [1]. These fates are associated with differential chemoattractant receptor expression profiles. The GC precursors likely following a CXCL12/13 gradient migrate from the follicle edge to the follicle center to form a nascent GC. Maturing GCs develop distinct anatomic regions, the light and dark zones, populated by B cells termed centroblasts and centrocytes, respectively. This segregation depends in part upon differential sensitivity of the cells to the chemokines CXCL12 and CXCL13 [5]. To generate highly mutated antigen receptors and to select B cells bearing high affinity antigen receptors, B cells recycle between these zones [6], [7], [8]. The decision to recycle is controlled by light zone helper T cells, which select light zone B cells based on their ability to acquire and present antigen [9]. Those B cells not returning to the dark zone either die *in situ* or leave the GC differentiating into memory B or plasma cells. The mechanisms controlling the directed migration of B cells between these GC zones and eventually out of GCs remain largely enigmatic. A model of GC B cell migration based on differential chemoattractant receptor signaling requires a rapid decline in B cell chemokine sensitivity following zonal transition to maintain discrete dark and light zones [10].

The sensitivity of B cells to chemokines can be rapidly modulated by two basic mechanisms: uncoupling the receptor from second messengers or by attenuating second messenger signaling [11], [12]. RGS proteins affect chemoattractant receptor signaling via the later mechanism. Chemoattractant receptors largely use the G_i_ subfamily of heterotrimeric G-proteins as signal transducers [13], [14]. Ligand engagement of chemoattractant receptors typically results in receptor/heterotrimeric G-protein coupling, Gα_i_ subunit GDP-GTP exchange, Gα_i_ dissociation from Gβγ, downstream effector activation, and directed migration. Since Gα_i_ subunits possess an intrinsic GTPase activity, GTP hydrolysis facilitates re-assembly of heterotrimeric G-protein causing signaling to cease. By dramatically accelerating the intrinsic GTPase activity of Gα_i_ subunits, RGS proteins reduce the duration that Gα_i_ subunits remains GTP bound, thereby decreasing effector activation [11], [15]. Either altering the expression or availability of RGS proteins to Gα_i_, would provide a mechanism to control the sensitivity of GC B cells to chemoattractants.

One RGS protein prominently expressed by GC B-lymphocytes and lymphomas of a GC origin is RGS13 [16]. Consistent with a role for RGS13 in regulating the B cell responses to chemoattractants, reducing *RGS13* expression in a human B cell line enhanced the magnitude and duration of chemokine receptor signaling while overexpression led to the opposite phenotype [17]. *Rgs13* is also expressed by mast cells and similar to the results with B cells, a mast cell line knock-down enhanced chemoattractant signaling [18]. Although RGS13 is among the smallest of the RGS proteins, essentially an RGS domain with a small N-terminus, RGS13 has additional biochemical roles mediated by interactions of its N-terminus with other proteins. In mast cells its N-terminus interacts with the regulatory p85α subunit of phosphatidylinositol-3-OH kinase disrupting the FcεRI-activated scaffolding complex [19]. Its N-terminus can also form a complex with the transcription factor CREB. Increased cAMP or Ca^2+^ signaling promotes the translocation of RGS13 into the nucleus where it binds phosphorylated CREB and core binding protein (CBP)/p300. This reduces CREB mediated transcription [20]. Suggesting that this may be important for B cell function, CREB signaling has been shown to control a genetic program that promotes GC B cell proliferation and self-renewal while inhibiting GC B cell differentiation [21].

To examine the functional importance of *Rgs13* expression in GC B cell chemotaxis and GC zoning, and to determine the impact of the loss of RGS13 inhibition of CREB mediated transcription in B cells, we generated mice in which the *Rgs13* coding region was replaced with green fluorescent protein (GFP). Using these mice we carefully documented *Rgs13* expression using GFP as a surrogate marker both *in vivo* using intravital microscopy and *in vitro* using flow cytometry. We examined the consequences of a lack of RGS13 on GC organization, GC B cell chemotaxis, and T cell dependent antibody responses. We found an expanded mucosal GC compartment and an exuberant antigen induced splenic GC response populated by B cells that exhibited a surprisingly altered gene expression profile. The implications of our results are discussed.

## Materials and Methods

### Animals

The *Rgs13*GFP KI mouse was generated by replacing exon 3 of the *Rgs13* gene with the coding region for enhanced GFP gene (Figure S1). The mice were generated on a C57BL/6 background by Ozgene (Bentley, Australia) and maintained both as heterozygotes and homozygotes. C57BL/6 and C57BL/6 CD45.1 (B6. SJL-Ptprc^a^Pep3^b^/BoyJ) mice were purchased from Jackson Laboratory (Bar Harbor, Maine). The mice were housed under specific pathogen-free conditions. All procedures were approved by and performed in accordance with the guidelines from the Institutional Animal Care Committee at the National Institutes of Health. When Jackson C57/BL6 mice were used as controls they were age and sex matched to the KI mice and co-housed for a minimum of two weeks. Littermate controls were used to verify germinal center formation and all studies involving Peyer's patches otherwise co-housed C57Bl/6 mice were used as controls.

### Flow Cytometry and antibodies

Single cells were re-suspended in PBS plus 2% FBS and stained with fluorochrome-conjugated or biotinylated antibodies against B220 (RA3-6B2), IgD (11-26c-2a), IgM (R6-60.2), CD24 (M1/69), CD3 (145-2C11), CD4 (GK1.5), CD5 (53-7.3), CD8 (53-6.7), CD11c (HL3), CD11b (M1/70), CD138 (281-2), CD19 (1D3), CD38 (90), IgG1 (X56), CD93 (AA4.1), BP-1 (6C3), GL-7 (GL-7, Ly-77), CD95 (Jo2), CD21 (4E3), CD23 (B3B4), CD43 (S7), CD184 (CXCR4, 2B11), CXCR5 (2G8), CCR7 (4B12), CD69 (H1.2F3), CD86 (GL1), CD279 (PD-1, RMP1-30), CD45.1 (A20), CD45.2 (104) (all from eBioscience or Biolegend, San Diego, CA; or BD Pharmingen, San Jose, CA). Biotin-labeled antibodies were visualized with fluorochrome-conjugated streptavidin (eBioscience). LIVE/DEAD® Fixable Aqua Dead Cell Stain Kit (Molecular Probes®, Grand Island, NY) was used in all experiments to exclude dead cells. Data acquisition was done on FACSCanto II (BD) flow cytometer and analyzed with FlowJo software (Tree Star, Ashland, OR). The absolute number of live cells in tissues was estimated as follows: total cell count  =  (detected live gate singlet cell number) × ((total volume of tube)/(detected flow time (sec)) x dilution factor. Flow rate of FACS CANTOII is 60 µl/min at medium speed.

### Cell proliferation

The cell proliferation studies were performed using the cell Proliferation Dye eFluor® 450 (eBioscience) in a standard dye dilution assay. Purified B cells were stimulated with various combinations of the indicated reagents: 1 μg/ml CD40 (HM40-3, BD, San Jose CA), 1 μM terbutaline, 10 nM prostaglandin E2 (PGE2) (both from Sigma, St Louis MO), recombinant BAFF (100 ng/ml), IL-4 (10 ng/ml), IL-17 (20 ng/ml), and IL-21 (20 ng/ml) for 72 h. All the recombinant proteins were from R&D Systems, Minneapolis MN). Data acquisition was done on FACSCanto II (BD) flow cytometer. Proliferation indexes were calculated using FlowJo software (Tree Star), which is defined as the total number of divisions divided by the number of cells that went into division, assuming no cell death.

### Mixed chimeras and cell sorting

Mixed chimeric mice were made by reconstituting irradiated CD45.1 mice with a 1∶1 mix of bone marrow from C57BL/6 CD45.1 mice (WT) and from KI (CD45.2). The success of each bone marrow engraftment was checked by sampling blood of the recipient mice 28 days later. No obvious bias was noted between the different sources of bone marrow cells in their ability to reconstitute the mice was noted. The mice were used 6–8 weeks after reconstitution. The mixed chimeric mice were immunized with sRBCs via intraperitoneal injection and 8–10 days later the splenocytes were isolated and immunostained with GL7, CD45.1, CD45.2, CD95, CD38, and B220 Abs. The cells were sorted for B220^+^GL7^+^, CD95^+^CD38^−^ and either CD45.1^+^ (WT) or CD45.2^+^ (KI) by FACSAria flow cytometer (Becton Dickinson). The CD45.2 GC B cells were greater than 90% GFP positive.

### Chemotaxis assays

Murine CCL19, CXCL12, and CXCL13 were purchased from R&D Systems. Cells were purified from spleen, LN, and Peyer's patches using standard protocols and immunostained for B220, GL7, CD38 and CD95 (spleen and Peyer's patch) and with B220 and IgD (LN). Migration assays were performed using a Transwell chamber (Costar Corp., Corning, NY), as previously described [22]. Each condition was performed in triplicate wells. Cells were added in a volume of 100 µl to the upper wells of a 24-well Transwell plate with a 5 µm insert. Lower wells contained 600 µl of assay medium with various concentrations of chemokines. The number of cells that migrated to the lower well following two hours of incubation at 37°C were counted using a FACS Canto II (BD Biosciences) or a MACSQuant flow cytometer (Miltenyi Biotec). Specific migration was calculated by taking the difference between the numbers of transmigrating cells of a given subset in the presence of chemoattractant from those where no chemoattractant was present, dividing by the total number of cells of that subset in the starting cell suspension, and multiplying the results by 100. Graphs were generated using Prism (Graphpad Software).

### Immunizations, ELISA, and ELISPOT

WT and KI mice were immunized with either sRBCs or TNP-KLH. For the sRBC immunizations 200 µl of 10% solution of sRBCs (Division of Veterinary Resources, NIH) was given by intraperitoneal injection. In some instances TNP-KLH (Biosearch Technology, Novato, CA) mixed with Imject® Alum (Thermo Scientific, Rockford, IL) was introduced to mice (100 μg) via intraperitoneal injection or injected intradermally (50 μg). Mice were boosted with same dose of antigen at the indicated days along with Alum. Serum TNP specific Ig levels in these mice were analyzed by ELISA. Briefly, 96 well ELISA plates (Nunc, Napersville, IL) were coated with TNP_3_-BSA or TNP_34_-BSA (Biosearch Technology) overnight at 4°C, washed and blocked with 5% BSA fraction V (Sigma, St Louis MO), serum titers were then added to the plates, and the plates incubated 4 h at 4°C. After washing alkaline phosphatase-labeled goat anti mouse Ig isotype specific antibodies were added for 2 h at RT (SouthernBiotech, Birmingham, Alabama). After washing, PNPP one component substrate (SouthernBiotech) was used to detect the amount of secondary antibody bound. For the ELISPOT analysis a standard protocol was used. Briefly, various dilutions of single cell suspensions prepared from bone marrow, spleen, and blood were cultured in plates previously coated with TNP-BSA or goat antibodies specific for mouse Ig. The cells were incubated for 3 h at 37°C and then washed with Tween/PBS to remove the cells. Next the plates were incubated with alkaline phosphatase-labeled goat anti-mouse isotype specific antibodies for 2 hours, washed, and nitro-blue tetrazolium and 5-bromo-4-chloro-3′-indolyphosphate were used to detect the ELISPOTs. ELISPOTs were counted manually and in some instances quantitated with an ELISPOT reader (Cellular Technology Limited, Shaker Heights, Ohio).

### Immunohistochemistry and immunocytochemistry

Freshly isolated spleens from mice were snap frozen in Tissue-Tek OCT compound (Sakura Finetek, Torrance, CA). Frozen OCT splenic sections (7 μm) were acetone fixed for 2 min, and dried at room temperature. Slides were rehydrated in Tris-buffered saline (TBS) and stained in a humidified chamber in TBS/0.1% BSA/1% mouse serum overnight at 4°C or 1 h. For immunohistochemistry primary antibodies included hamster anti-mouse CD3 (145-2C11, purified), rat anti-mouse IgD (11-26c.2a, purified), rat anti-mouse CD35 (8C12, biotinylated), rat anti-mouse CD45R/B220 (RA3-6B2, purified) all from BD Pharmingen. Donkey anti-mouse IgG (H+L) alkaline phosphatase and goat anti-mouse IgM F(ab')_2_ alkaline phosphatase were purchased (Jackson ImmunoResearch Laboratories). Anti-Ki67 (purified; rabbit polyclonal) was from Abcam, and anti-RGS13 rabbit polyclonal was previously described [16] and a gift from Dr. Kirk Druey (NIAID, NIH). Biotinylated antibodies were detected with streptavidin-alkaline phosphatase (Jackson ImmunoResearch Laboratories), and purified mAbs with alkaline phosphatase or Horseradish peroxidase conjugated goat anti-Armenian hamster IgG (H +L), donkey anti-rabbit IgG (H+L), donkey anti-rat IgG (H+L), or goat anti-rabbit IgG (H+L), (Jackson ImmunoResearch Laboratories). Horseraddish peroxidase was reacted with DAB (Peroxidase Substrate Kit; Vector, Burlingame, CA), and alkaline phosphatase with Fast Blue/Napthol AS-MX phosphate (Sigma-Aldrich). Peroxidase Block (Dako, Carpinteria, CA) or Levamisole (Vector Labs) were used to block endogenous alkaline phosphatase and peroxidase activities, respectively. Slides were mounted using Crystal Mount (Electron Microscopy Sciences, Hatfield, PA). For immunocytochemistry, frozen acetone fixed slides were stained with a mixture of CD45R/B220 (RA3-6B2, Alexa Fluor 488, BD Pharmingen), and rabbit anti-RGS13 4C overnight. The RGS13 antibody was detected with Alexa Fluor 568 IgG (H + L) from Invitrogen. Slides were mounted with Vectashield (Vector Labs). Images were acquired either with an Olympus BX-50 microscope equipped with a ProgRes-digital microscope camera or a Zeiss Axiovert 200 fluorescent microscope equipped with a Sensicam EM camera.

### RNA isolation and Real-time Quantitative PCR

RNA was isolated with TRIZOL Reagent (Invitrogen, Grand Island, NY) according to the manufacturer's instructions. Complementary DNA (cDNA) was synthesized from 1 μg RNA with Omniscript RT Kit (QIAGEN, Valencia, CA). Some of the real-time PCR primers used to amplify genes are listed below while those for *Mta3*, *Creb1*, *Crebbp*, *EP300*, *Stk11*, *Cdkn1b*, *Bcl6*, *Smarca2*, *Trp53*, *Prdm1*, *Rgs2* were from Qiagen (QuantiTect primer QT01075249, QT00174104, QT01055775, QT01052205, QT00138117, QT01058708, QT01057196, QT01052632, QT00101906, QT00106512, and QT00122689, respectively). Real-time PCR was performed using a 7500 Real-Time PCR System (Applied Biosystems, Carlsbad, CA) following the Rotor-Gene SYBR Green PCR kit (QIAGEN, Valencia, CA) protocol. The *Rgs13* and *Rgs1* primers/probes were Mn00450170_m1 & Mn00462629_m1 (Applied Biosystems, Carlsbad, CA) and used on the 7500 Real Time PCR System (Applied Biosystems). The endogenous control was *Gapdh* detected using a VIC/MGB Probe (4352339E, Applied Biosystems). The results were normalized to the expression levels of the GAPDH reference gene and the relative mRNA levels were calculated using the 2^−ΔΔCt^ method. Quantitative PCR of mouse

*Crtc2* Product length 71 bp: Forward primer GGCCTTCGAGGAGGTGATG

Reverse primer TATAAGCCAGTCGCAGTTTTTGG

*Ccnb2* Product length 475 bp: Forward primer GGAAGAAACTGCAGCTGGTC

Reverse primer GCGATGAACTTGGTACGGTT

*Ccna2* Product length 285 bp: Forward primer AGAGGCAGCCAGACATCACT

Reverse primer GCTCCATTCTCAGAACCTGC

*Aicda* Product length 285 bp: Forward primer GATGGATGCCAACACGGTTAAACA

Reverse primer AAGCGTCATTTCCTTGCCACGGTC

*Gcet2* Product length 375 bp: Forward primer ATGGGGAACTGTTTGCAGAG

Reverse primer ATCTGTGGAAGGTAGGGGCT.

### Immunoblotting

A polyclonal antibody against RGS13 has been previously described [16] and anti-Actin (Sigma) was used for immunoblotting. Spleen cell lysates from immunized WT and KI mice were prepared using RIPA buffer containing 1 mM NaF, 1 mM PMSF, 1 mM DTT, and 1 mM Na_3_VO_4_ with Complete and PhosStop (Roche Diagnostics, Indianapolis, IN) protease and phosphatase inhibitor cocktail tablets. The cell lysates were centrifuged at 20,000×g for 10 min at 4°C, and equivalent amounts of protein were loaded onto 10% NuPage Bis-Tris gels (Invitrogen). After electrophoresis, the proteins were transferred to nitrocellulose membranes. The membrane was first incubated in Tris-buffered saline-Tween (TBST) [20 mM Tris-HCl (pH 7.6), 133 mM NaCl, and 0.1% Tween] with 5% milk for 1 hour. The membrane was washed once with TBST, and the first antibody was added to the membrane in TBST containing 5% milk overnight at 4°C. The membrane was washed four times for 10 min with TBST, incubated with the appropriate horseradish peroxidase (HRP)-conjugated secondary antibody for 1 h, washed six times for 10 min in TBST, and subjected to enhanced chemiluminence, which was detected with HyBlot CL film (Denville Scientific Incorporation, Metuchen, NJ).

### Intravital two-photon laser scanning microscopy (TP-LSM)

Inguinal LNs were prepared for intravital microscopy as described [23], [24]. Cell populations were labeled for 15 minutes at 37°C with 2.5∼5 mM red cell tracker CMTMR (Molecular probes), blue cell tracker CMF_2_HC (Molecular probes) or eFluor® 670 (eBioscience). 10–30 million labeled cells of each population in 200 ml of PBS were adoptively transferred by tail vein injection into 6∼10-week-old recipient mice. After anesthetizing the mice by intraperitoneal injection of Avertin (300 mg/kg, tribromoethanol, Sigma), the skin and fatty tissue over inguinal LN were removed. The mouse was placed in a pre-warmed coverglass chamber slide (Nalgene, Nunc). The chamber slide was then placed into the temperature control chamber on the Leica SP5 microscope. The temperature of air was monitored and maintained at 37.0±0.5°C. Inguinal LN was intravitally imaged from the capsule over a range of depths (10–220 µm). Two-photon imaging was performed with a Leica SP5 inverted 5 channel confocal microscope (Leica Microsystems) equipped with 20× multi-immersion objective, 0.7 NA (immersion medium used 80% glycerol) or 25× water dipping objective, 0.95 NA (immersion medium used distilled water). Two-photon excitation was provided by a Mai Tai Ti:Sapphire laser (Spectra Physics) with a 10 W pump, tuned to 810 or 910 nm. Emitted fluorescence was collected using a 4 channel non-descanned detector. Wavelength separation was through a dichroic mirror at 560 nm and then separated again through a dichroic mirror at 495 nm followed by 460/50 nm emission filter for second harmonics or CMF_2_HC; 525/50 emission filter for CMFDA (Molecular probes) and GFP; a dichroic mirror at 650 nm followed by 610/60 nm emission filter for CMTMR and phycoerythrin conjugated antibodies; and the eFluor® 670 signal was collected by 680/50 nm emission filter. Sequences of image stacks were transformed into volume-rendered four-dimensional videos using Imaris software v.7.5.0 or v.7.5.2 (Bitplane), and the spot analysis was used for semi-automated tracking of cell motility in three dimensions.

### Statistics

In vivo results represent samples from 3 to 6 mice per experimental group. *In vitro* results represent mean values of quadruplicate samples. SEM and p values were calculated with the Mann-Whitney test or unpaired t tests using Microsoft Excel 2011 or GraphPad Version 5 Prism software.

## Results

### GC B cells express *Rgs13* and generation *of* the *Rgs13*GFP KI mice

**Figure 1:**
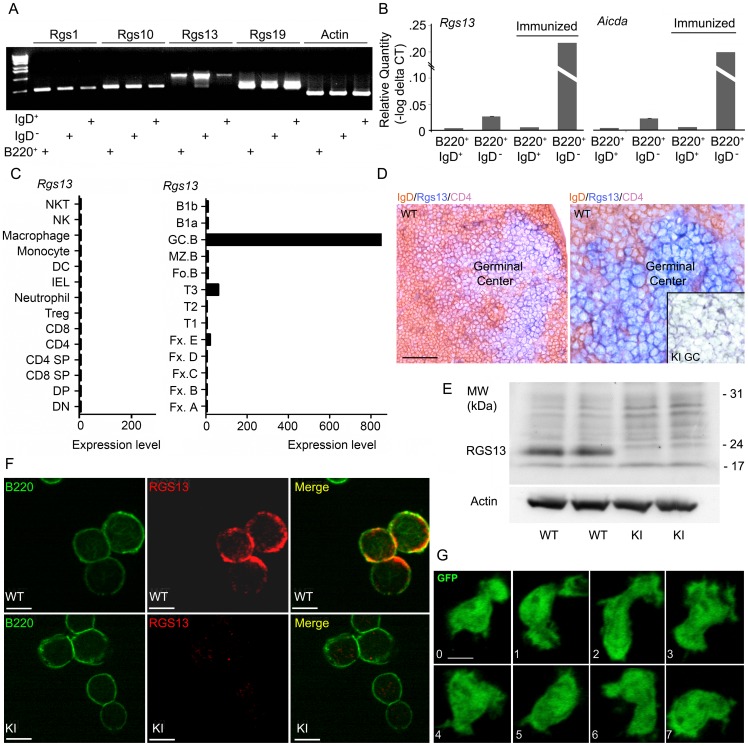
RGS protein gene expression in various B cell populations. A. RT-PCR analysis of RNA extracted from sorted B cell population obtained from mice 8 days after sRBC immunization. B. Quantitative RT-PCR analysis of sorted B cell populations from immunized and non-immunized mice. C. Microarray data analysis of *Rgs13* expression in sorted cell populations. Data extracted from the Immunological Genome Project database (http://www.immgen.org/databrowser/index.html) D. Brightfield microscopy of a sectioned Peyer's Patch prepared from WT and KI mice Immunostained for RGS13, CD4, and IgD. Scale bar, 50 µm, left panel; and 3X zoom, right panel. An insert in the right panel is from the GC region of a similarly immunostained KI mouse. E. Immunoblot analysis of RGS13 expression in WT and KI immunized splenocytes prepared from day 10 immunized WT and KI mice. Actin levels were used as a loading control. F. Confocal microscopy of Peyer's Patch cells prepared from WT and KI mice immunostained for RGS13 and B220. Individual and merged images are shown. Scale bar, 7 µm. G. Confocal microscopy of GFP positive B cell prepared from D7 immunized KI mice.

Previous results had indicated that *Rgs13* is expressed in mouse GCs [16]. We confirmed these results by examining *Rgs13* expression in sorted B220^+^IgD^+^, B220^+^IgD^−^, and B220 positive cells from the spleens of mice immunized 8 days previously with sheep RBCs (sRBCs, Figure 1A). Using quantitative real time RT-PCR *Rgs13* and *Aicda* exhibited a similar pattern of expression in various B cell populations including marked enrichment in the B220^+^IgD^−^ population (Figure 1B). When we queried the Immunological Genome Project microarray database *Rgs13* expression nearly exclusively resided in the GC B cell population (Figure 1C). Among the genes in the database *Rgs13* expression best correlated with that of *Mybl1* (.985), *Aicda* (.985) and *GM600* (.983). *Mybl1* and *GM600* are also strongly expressed in GC B cells. Despite the strong GC B expression of *Rgs13*, examination of the promoter regions of both the human and mouse *Rgs13* genes did not identify an obvious set of transcription factor binding sites that might account for its GC B cell expression (J. Kehrl, unpublished observation).

To examine the impact of the loss *Rgs13* for B cell function, we generated mice in which the *Rgs13* coding sequence was replaced with that for green fluorescent protein (GFP). The gene targeting strategy is shown (Figure S1). The KI did not adversely affect embryonic development and the mice thrived and bred normally (data not shown). As expected the targeting disrupted *Rgs13* mRNA expression. In addition, it modestly affected other RGS protein expression as we noted increases in *Rgs1*, *Rgs10*, and *Rgs19* in RNA extracted from Peyer's Patches (Figure S1). WT and KI mice had similar lymphocyte populations in immune organs of non-immunized 6–8 week mice with minor exceptions (data not shown and Figure S2). We noted an expansion of B cells in Peyer's patches and some minor changes in bone marrow B cell development. Overall the number of B220^+^ cells in the bone marrow lymphocyte gate was slightly reduced in the KI mice (74±5% versus 83±6%). Immunostaining Peyer's patches from WT and KI mice for RGS13 demonstrated immunoreactivity in the GC region in WT but not in KI mice (Figure 1D).

Immunoblotting confirmed the loss of RGS13 expression in spleen cells prepared from immunized mice (Figure 1E). Immunocytochemistry revealed RGS13 expression in a subset of B220^+^ cells isolated from the spleen of an immunized mouse, while a similar B cell preparation from the KI mouse lacked immunoreactivity (Figure 1F). RGS13 immunoreactivity in the WT B cells was predominately located at plasma membranes however some nuclear expression was noted. A subset of cells isolated from Peyer's Patches of KI mice expressed GFP and exhibited a polarized and very dynamic behavior by live cell confocal microscopy (Figure 1G). These results confirm the expression of RGS13 in GC B cells and demonstrate the GFP expression in a subset of B cells in the KI mice

### Immunization of the *Rgs13*GFP KI mice results in the rapid induction of GFP in a subset of B cells and robust early B cell responses

**Figure 2:**
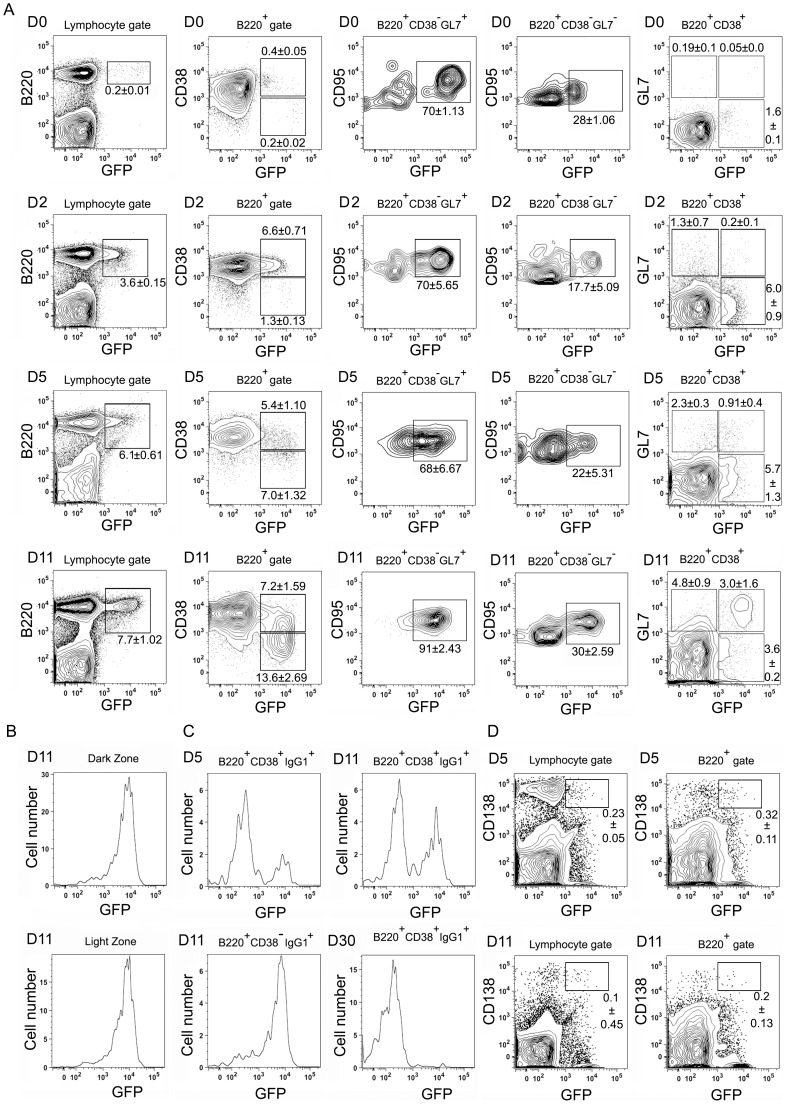
Flow cytometric analysis of GFP expression in immunized *Rgs13*GFP KI mice. A. Representative flow cytometry plots of spleen cells prepared from KI mice prior to and at days 2, 5, and 11 post sRBC immunization using antibodies specific for B220, CD38, CD95 (FAS), and GL7 along with GFP. Cells were gated as indicated above each plot and GFP expression is shown on the x-axis. B. Representative flow cytometry plots of spleens cells prepared from KI mice at day 11 post immunization using the same antibodies as part A along with antibodies specific for CXCR4 and CD86. Dark zone cells are GC B cells that are CXCR4^hi^CD86^low^ while light zone cells are CXCR4^low^CD86^hi^. C. Representative flow cytometry plots of spleens cells prepared from KI mice 5, 11, or 30 days post sRBC immunization using antibodies specific for B220, CD38, and IgG1 along with GFP. Cells gated as indicated. D. Representative flow cytometry plots of spleen cells prepared from KI mice at 5 or 11 days post immunization using antibodies specific for B220 and CD138 along with GFP. Cells gates are as indicated. All experiments preformed a minimum of 3 times with 2–4 mice and presented as the mean ± SEM of 2–4 mice per group.

We used flow cytometry to analyze GFP expression in KI mice lymphocytes following intraperitoneal (I.P.) immunization with sRBCs. Prior to immunization only 0.2% of the cells in the lymphocyte gate from KI mice expressed GFP and all were B220^+^. A few strongly GFP positive cells resided in the GC B cell gate (B220^+^CD38^−^GL7^+^CD95^+^). Some weaker GFP positive cells were found among the B220^+^CD38^+^ subset (Figure 2A, top panel). The expression of GL7 on B220^+^CD38^+^ cell may delineate precursors of memory and GC B cells [25]. Although these cells are uncommon in the unimmunized mice, in the KI mice some of them expressed GFP. Following immunization we noted that GFP expression rapidly increased in the KI B cells. At D2 post-immunization 6.6% of the B220^+^ cells expressed GFP and a small population of B220^+^ cells (1.6%) already exhibited a full GC phenotype. Among these GC B cells 70% expressed GFP. The B220^+^CD38^+^GL7^+^ population had increased although the % of GFP^+^ cells in that subset had declined (Figure 2A, second panel). By D5 post-immunization 8.4% of the spleen B220^+^ cells had a full GC phenotype and 68% were GFP^+^. In the B220^+^CD38^+^GL7^+^ subset 28% expressed GFP (Figure 2A, third panel). Finally, by D11, 14.5% of the KI B cells had a full GC phenotype and 90% expressed GFP. In the B220^+^CD38^+^GL7^+^ subset 38% were GFP^+^ (Figure 2A, bottom panel). Next, we checked the expression of GFP in light and dark zone germinal center B cells. Dark and light zone B cells can be distinguished by their expression of CXCR4 and CD83 [9]. At D11 post-immunization we found that dark zone (CXCR4^high^CD83^low^) and light zone (CXCR4^low^CD83^high^) B cells expressed similar levels of GFP (Figure 2B).

*Rgs13* has been reported to be expressed in murine memory B cells [26], but not in human plasma cells as Blimp-1 (*Prdm1*) is known to repress its expression [27]. To test whether newly generated memory B or plasma cells express *Rgs13* we checked GFP expression in switched B cells and CD138^+^ cells at D5, D11, and D30 post immunization. A minority of day 5 B220^+^CD38^+^IgG1^+^ cells expressed GFP while 50% of similar cells at day 11 did. Nearly all the B220^+^CD38^−^IgG1^+^ cells at D11 post immunization expressed GFP. However, by D30 the B220^+^CD38^+^IgG1 positive cells lacked GFP expression indicating that long term switched memory B cells do not express high levels of Rgs13 (Figure 2C). Consistent with limited expression of *Rgs13* in plasma cell only a small percentage of the CD138^+^ cells analyzed 5 and 11 days post immunization expressed GFP (Figure 2D). *RGS13* has also been reported to be expressed in human follicular helper T cells, which acquire CXCR5 and migrate into the LN follicle to support GC B cells [28]. While we found a modest increase in the number of follicular helper T cells in the KI mice, we detected little or no GFP expression in cells gated for CD4, CXCR5, and PD-1 expression prepared from the spleens of immunized KI mice or from Peyer's patches (Figure S3). Together these results indicate that *Rgs13* expression is rapidly induced in a subset of B cells during the course of a T-cell dependent immune response. Eventually most mature dark and light zone GC B cells express *Rgs13*. Our results are consistent with an expression of *Rgs13* in early memory B cells followed by a reduction in mature memory B cells. In newly generated plasma cells *Rgs13* is rapidly downregulated.

**Figure 3:**
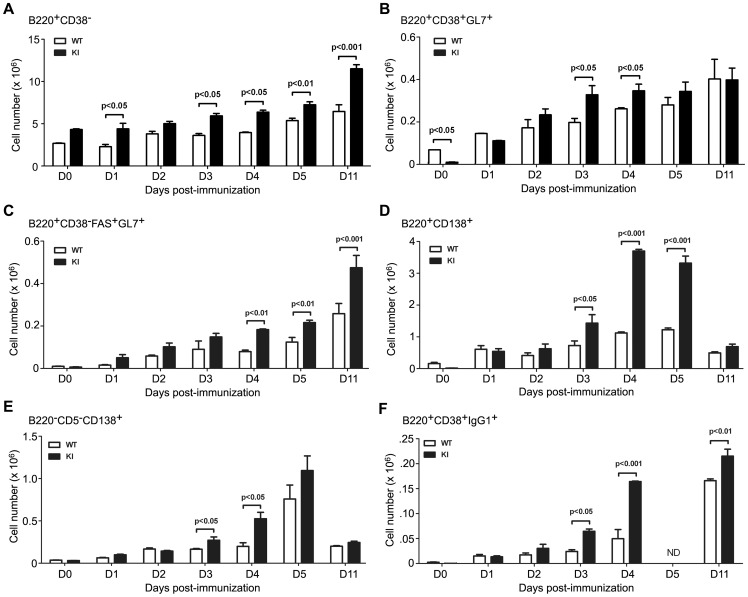
Increased numbers of GC, plasma, and memory B cells following immunization of the *Rgs13*GFP KI mice. A. Flow cytometric quantification of absolute number of B220^+^CD38^−^ cells in the spleens of WT versus KI mice at various days post sRBC immunization. B. Flow cytometric quantification of absolute number of B220^+^CD38^+^GL7^+^ cells in the spleens of WT versus KI mice at various days post sRBC immunization. C. Flow cytometric quantification of absolute number of GC B cells in the spleens of WT versus KI mice at various days post sRBC immunization. D. Flow cytometric quantification of absolute number of early plasma cells in the spleens of WT versus KI mice post immunization. E. Flow cytometric quantification of absolute number of mature plasma cells in the spleens of WT versus KI mice following immunization. F. Flow cytometric quantification of absolute number of B220^+^CD38^+^IgG1^+^ cells in the spleens of WT versus KI mice post immunization. Results are based on the analysis of 6 WT versus 6 KI mice at each time point. The absolute cell number of B-cell subsets & plasma cells (CD138^+^) were calculated from the flow cytometric analysis and presented as the mean ± SEM of six per group. Statistics are from unpaired t tests.

Next, we compared the kinetics of the appearance of activated B cells (B220^+^CD38^−^); non-GC memory cell and GC precursors (B220^+^CD38^+^GL7^+^); GC B cells (B220^+^CD38^−^CD95^+^GL7^+^); early switched memory B cells (B220^+^CD38^+^IgG1^+^); early plasma cells (B220^+^CD138^+^); and mature plasma cells (B220^−^CD5^−^CD138^+^) in the spleens of WT and KI mice immunized with sRBCs (Figure 3A–F). The number of B cells that had downregulated their expression of CD38 increased following immunization and was consistently higher in the KI mice (Figure 3A). The non-switched memory B cell and GC precursors defined by GL7 and CD38 expression [25] increased as consequence of immunization and the KI mice had more of these cells at D3/4 post-immunization (Figure 3B). The typical GC B cells increased in both the WT and KI mice following immunization and from D4 onward were statistically increased in the KI mice (Figure 3C). The number of early plasma cells progressively increased from D1–D5, but declined at D11. The KI mice had a clear increase at D3–D5 (Figure 3D). The mature plasma cells followed a similar kinetics, but the KI cells exceeded the WT only at D3/4 (Figure 3E). Finally, the number of IgG1 positive B cells that expressed CD38, making it unlikely that they had arisen from GC B cells, progressively increased with the KI having increased numbers at D3, D4, and D11. Overall we found these results rather surprising as the lack of *Rgs13* had resulted in an overall more robust B cell immune response evident 3–4 days after immunization. This suggested that *Rgs13* expression limits the early expansion of B cells that occurs during a T cell dependent immune response.

### *In vivo* visualization of GFP^+^ cells at the T-B border, the interfollicular zone, and in nascent GCs

**Figure 4:**
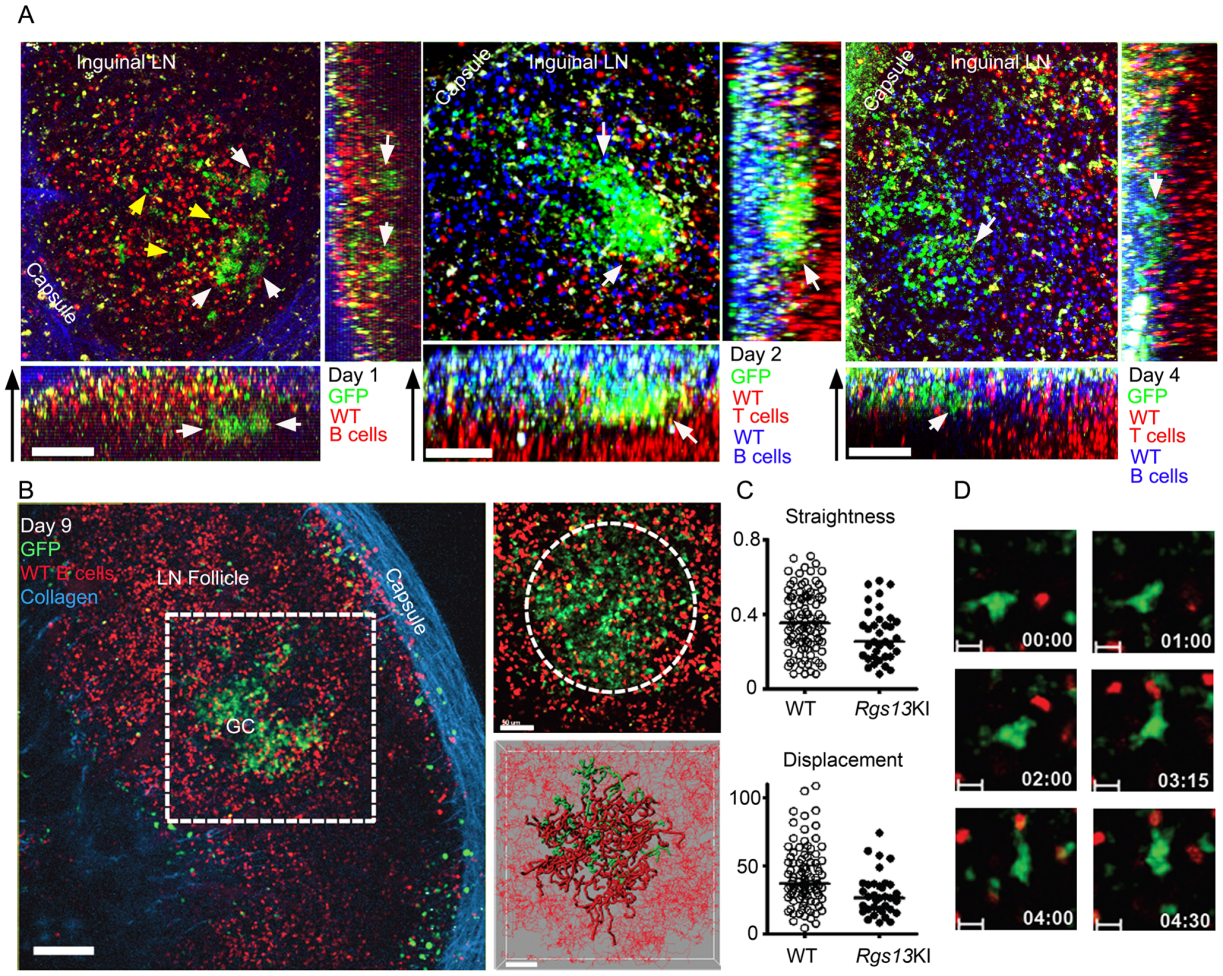
Rapid induction of GFP expression *in vivo* following immunization. A. Intravital TP-LSM of the inguinal LN of KI mice immunized with sRBCs 1, 2, or 4 days previously. The day prior to imaging labeled B cells or labeled B and T cells from non-immunized WT mice were transferred to outline LN follicle and T cell zone, respectively. Shown are X, Y; X, Z (below); and Y, Z (right) projections from an image stack collected on the indicated day. Clusters of GFP positive cells shown with white arrow and GFP positive cells in the follicle delineated by yellow arrows. B. Intravital TP-LSM of the inguinal LN of a KI mouse immunized with sRBC 7 days previously. The day prior to imaging splenic B cells from a non-immunized WT mouse were adoptively transferred to outline the LN follicle. The left image shows GFP expression, the transferred WT B cells (red), and collagen (blue). The middle two images show the region subjected to analysis and tracks of the WT B cells (red) and the endogenous GFP expressing cells present in the KI mice. C. Track analysis of GFP^+^ KI B cells in the GC region versus WT B cells within the follicle. Statistical significance of straightness and displacement was calculated by Mann Whitney test. (*; p<0.05, **; p<0.01) D. Electronically zoomed time lapse images from GFP^+^ KI B cell in the GC from part B intravital TP-LSM imaging.

Since GFP expression is rapidly induced in the spleens of KI mice following immunization and well expressed later in GC B cells, we checked whether we could visualize GFP positive B cells *in vivo* at various time points after immunization. Using intravital two-photon imaging we examined non-immunized mice and those that had received either sRBCs via intraperitoneal injection or a subcutaneous injection of TNP-KLH near the inguinal LN. The inguinal LNs of immunized mice were imaged 1, 2, 4, and 9 days later. The day prior to imaging we adoptively transferred differentially labeled WT B cells (D1 and D9) or both WT B and T cells (D2 and D4). The WT B and T cells helped delineated the follicle and T cell zone in the LN, respectively. Prior to immunization few if any GFP+ cells were visible near or in the LN follicle (data not shown). However even D1 post-immunization several clusters of GFP positive cells were easily discernible at the T-B cell border (Figure 4A, left panel; Video S1). This is best observed in the X-Z projection shown below. As we are observing all of the endogenous responding B cells and not just a limited number of transferred transgenic B cells delineation of individual cells within the clusters was difficult. We noted an occasional GFP positive cell already located in the follicle. By D2 GFP positive cells were much more numerous, located at the T-B border and penetrating into the follicle (Figure 4A, middle panel, Video S2). All of mice we imaged at day 1–2 post-immunization had visible of clusters of GFP positive cells located at the T-B border. By D4 the majority of GFP positive cells had disappeared from the interfollicular region and most now resided in the follicle (Figure 4A, right panel). On D9 the GFP positive cells outlined a typical GC. Within the GC we found a green haze along with an occasional clearly identifiable GFP positive cell (Figure 4B and Video S3). Tracking WT and GFP positive cells revealed a decreased displacement and straightness consistent with GC phenotype (Figure 4C). Individual cells had the morphology of typical GC B cells being large with dynamic membrane extensions (Figure 4D). These results indicate that *Rgs13* expression begins within a day of activation as GFP positive cells appear early after immunization, along the T-B border, and in the interfollicular regions. Within several days of immunization the GFP positive cells move towards and into the center of the LN follicle to establish a GC.

### Loss of Rgs13 leads to large GCs

**Figure 5:**
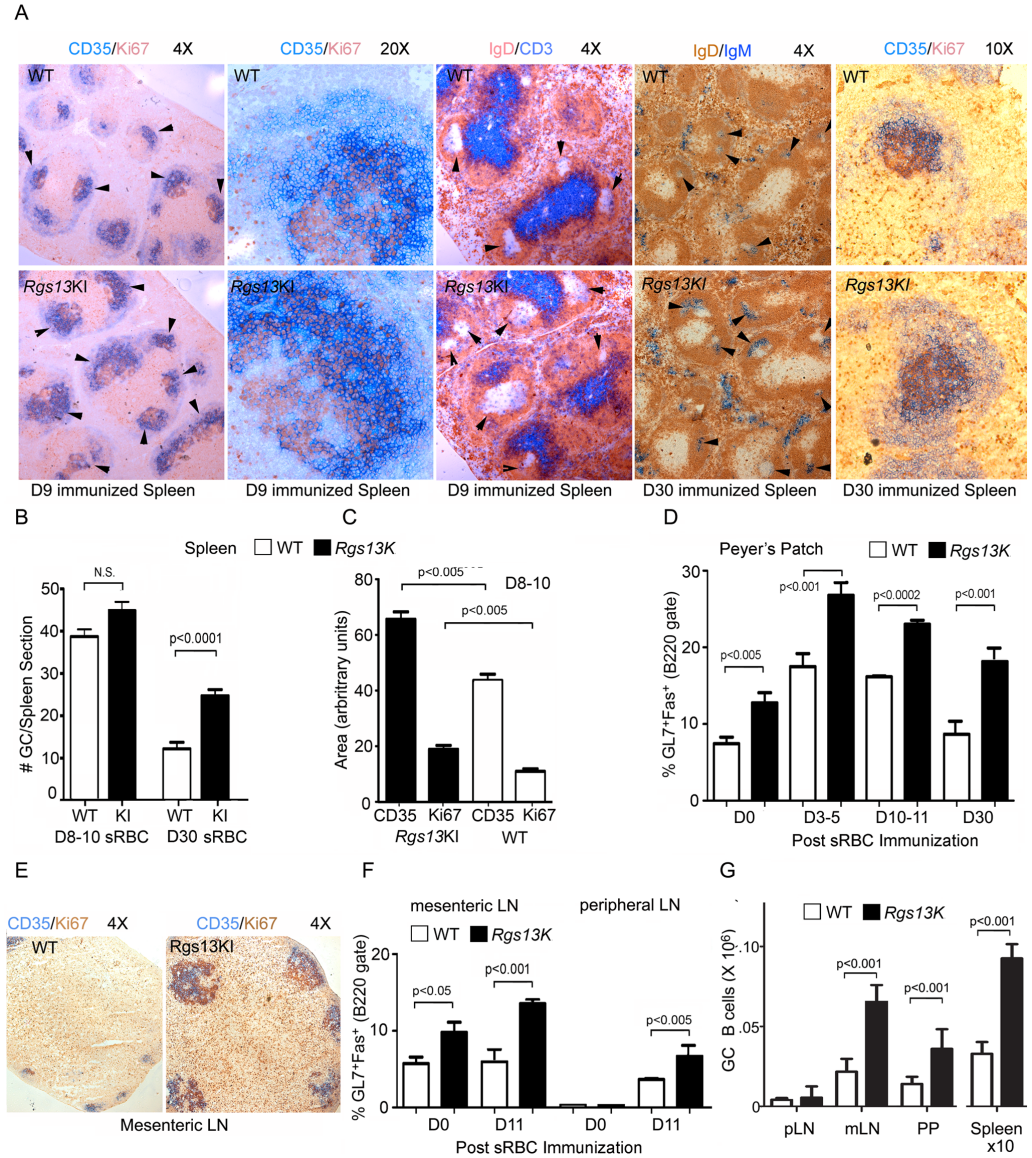
Large GCs in *Rgs13*GFP KI mice. A. Brightfield microscopy of representative spleen sections from day 9 and 30 sRBC immunized WT and KI mice using antibodies against CD35 and Ki67, IgD and CD3, or IgM and IgD. In some sections GCs are indicated with arrowheads. B. Quantification of the number of GCs per spleen section from WT and KI mice 8–10 or 30 days post-immunization with sRBCs. Eight WT and 8 KI mice (8–10 day) and 4 WT and 4 KI mice (day 30) immunostained for Ki67 and CD35 were used. Data is mean ± SEM. Statistics, unpaired t test. C. Quantification of CD35 and Ki67 immunostaining of individual GCs from WT and KI spleen sections prepared from 8–10 day post immunized animals. Data is mean ± SEM of the areas from CD35 and Ki67 immunostaining of 50 WT and KI GCs (unpaired t test). D. Flow cytometric analysis of B220^+^CD38^−^GL7^+^CD95^+^ cells in Peyer's patches from WT and KI mice prior to and post sRBC immunization. Data is % of B220 gate and is the mean ± SEM of 8 v. 8, 11 v. 11, 8 v. 8, and 4 v. 4 WT and KI mice at 0, 3–5, 10–11, and 30 days post immunization, respectively. Results compared by unpaired t test. E. Brightfield microscopy of representative mesenteric LNs from WT and KI mice using antibodies against CD35 and Ki67. F. Flow cytometric analysis of B220^+^CD38^−^GL7^+^CD95^+^ cells in mesenteric and peripheral LNs from WT and KI mice prior to and post sRBC immunization. Data is % of B220 gate and is the mean ± SEM of 4 WT v. 4 KI mice at each time point. Results compared by unpaired t test. G. Flow cytometric enumeration of B220^+^CD38^−^GL7^+^CD95^+^ cells in peripheral LNs (pLN), mesenteric LNs (mLN), Peyer's patches (PP) and the spleen from chimeric mice (CD45.1 versus CD45.2) 9 days post sRBC immunization. Data is mean ± SEM of cells recovered from 4 chimeric mice. Results are compared by unpaired t test.

The spleen morphology and the B and T cell regions were unperturbed in the sections prepared from the unimmunized KI mice (data not shown). However, upon sRBC immunization the GCs in the spleen sections prepared from the KI mice were noticeably larger than those observed in the sections from WT mice (Figure 5A). GCs were visualized by CD35 versus Ki67 staining. CD35 recognizes CR1 and identifies follicular dendritic cells (FDC) in the primary and secondary follicles and strongly reacts with FDCs in the GC light zone [5]. The Ki-67 protein (also known as MKI67) is a cellular marker for proliferation and can be used to delineate the dark zone region. In the KI mice spleens the light zone and dark zone regions were less distinct compared to those of WT mice. The Ki67 stained cells were less clustered into the dark zone and more prevalent in the light zone. At D30 post immunization the residual GCs appeared larger and more numerous than in the WT mice (Figure 5A). The numbers of GCs per spleen section from immunized WT and KI mice at D8-10 were similar while at D30 there was an increase in the KI mice (Figure 5B). The sizes of the GCs differed between the WT and KI mice spleens. Spleen sections immunostained with CD35 and Ki67 obtained D8-10 post- immunization revealed a 50% and 70% average increase, respectively, in the area reactive with the two antibodies in the KI mice (Figure 5C). Thus, the increase in CD38^−^GL7^+^CD95^+^ B cells noted by flow cytometry results predominately from larger not more numerous GCs at D8-10 post immunization.

While C57/BL6 mice from a clean mouse facility have relatively few spontaneous GCs in their spleens, constitutive GC formation occurs in Peyer's patches and mesenteric LNs as a result of continuous B cell stimulation by commensal bacteria. Examining the Peyer's Patches from the WT and KI mice revealed more GC B cells in the KI versus the WT (Figure 5D). Following sRBC immunization the % of GC B cells increased in both the WT and KI mice, but the difference between the WT and KI mice persisted (Figure 5D). The number of GC B cells in mesenteric LNs behaved in a similar fashion (Figure E & F). Peripheral LNs had few GC B cells unless immunized. Finally, we examined 1∶1 mixed bone marrow chimeric mice (WT and KI bone marrow) 3 months post reconstitution and D9 after sRBC immunization. These mice had a 2–3 fold greater expansion of the KI GC B cells compared to WT GC B cells in the spleen, mesenteric lymph node, and Peyer's patches although we did not detect a significant difference in peripheral LNs (Figure 5G). The KI GC B cells preferentially increased as a percentage of the follicular B cells in the chimeric mice. For example, 2% of the WT B220^+^ cells had a GC phenotype while 7% of the B220^+^ KI cells did so (results from the analysis of the spleens from 6 immunized chimeric mice). Together these results indicate that RGS13 helps organize GC morphology and limits the size of germinal centers.

### GFP expression marks GC B cells from the *Rgs13*GFP KI mice as being poorly responsive to chemokines

**Figure 6:**
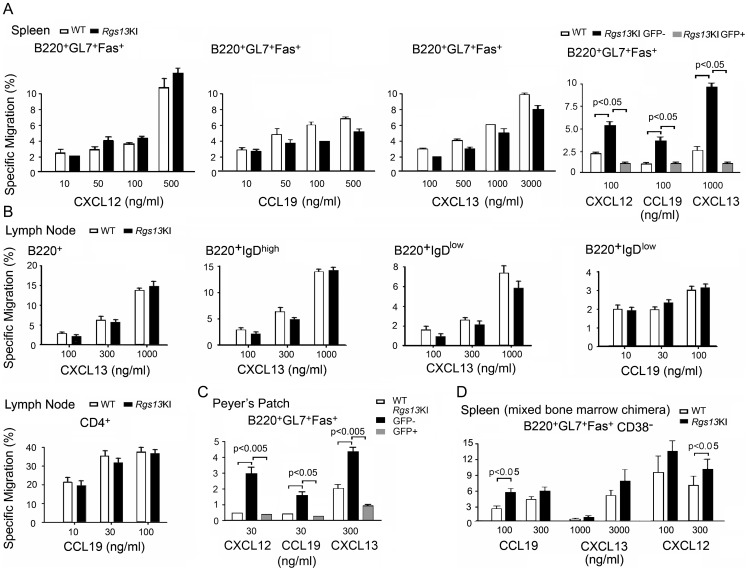
GC B cells from WT and *Rgs13*GFP KI mice exhibit similar responses to chemokines. A, Chemotaxis assays of WT and KI spleen cells from day 10 sRBC immunized mice immunostained with B220, GL7, and CD95 using various concentrations of CXCL12, CXCL13, or CCL19. In the last panel the KI GC B cells were further fractionated on the basis of GFP expression. Results are from the analysis of 4 WT and 4 KI mice with each assay preformed in triplicate. Data is mean ± SEM and statistics from unpaired t tests. B. Chemotaxis assays of WT and KI LN cells from D10 sRBC immunized mice immunostained with B220, CD4, and IgD using various concentrations to CCL19 or CXCL13. Results are from the analysis of 2 WT and 2 KI mice with each assay preformed in triplicate. Data is mean ± SEM. Experiment repeated twice with similar results. C. Chemotaxis assay of WT and KI Peyer's Patch cells immunostained with B220, GL7, and CD95 to indicated concentration of CXCL12, CXL13, or CCL19. The KI GC B cells were fractionated based on GFP expression. Results are from the analysis of 1 WT and 1 KI mice with each assay preformed in quadruplicate. Data is mean ± SEM and statistics from unpaired t test. Similar results in 3 other experiments. D. Chemotaxis assays of WT (CD45.2) and KI (CD45.1) spleen cells from day 10 sRBC immunized chimeric mice immunostained with B220, CD38, GL7, and CD95 using indicated concentrations of CXCL12, CXCL13, or CCL19. Results are from the analysis of 4 chimeric mice with each assay performed in triplicate. Data is mean ± SEM and statistics from unpaired t tests.

Many previous studies have found that GC B cells react poorly in standard chemotaxis assays [29], [30], [31]. One possible explanation for this is their high expression of RGS proteins. To test whether the loss of *Rgs13* expression affected murine GC B cell chemotaxis we prepared B cells from spleens of immunized WT and KI mice and tested the cells in standard chemotaxis assays using different concentrations of CXCL12, CXCL13, and CCL19. In contrast to our expectation we found no increased chemotaxis of the KI versus the WT splenic GC B cells (Figure 6A). However, if we fractionated the KI GL7^+^CD95^+^ B cells based on their GFP expression, the GFP^+^ cells performed much worse than did the GFP^−^ fraction (Figure 6A, last panel). This was not due to different levels of chemokine receptors as the levels of CXCR4, CXCR5, and CCR7 on GFP^+^ and GFP^−^ cells were similar (data not shown). The loss of *Rgs13* did not improve LN GC B or CD4 T cell responsiveness either (Fig. 6B). Similarly, GC B cells from Peyer's patches from KI and WT mice exhibited no significant differences (data not shown), but like the spleen GC B cells the GFP^−^ cells performed better than did the GFP^+^ cells (Figure 6C). Finally, we checked the chemotaxic responsiveness of GC B cells from mixed bone marrow chimeras, which allowed a more direct comparison of the WT and KI GC B cells. Here we did observe a slight increase in the specific migration of the KI GC B cells at some, but not at all chemokine concentrations (Figure 6D). Thus, while the *in vitro* migration assays could discern little difference between the WT and KI GC B cells, the lack of GFP expression in the KI GC B cells defined an interesting population of GC B cells that had a heightened responsiveness to chemokines.

### The *Rgs13*GFP KI mice have an augmented early antibody response, but a relatively normal affinity maturation of their antibody responses

**Figure 7:**
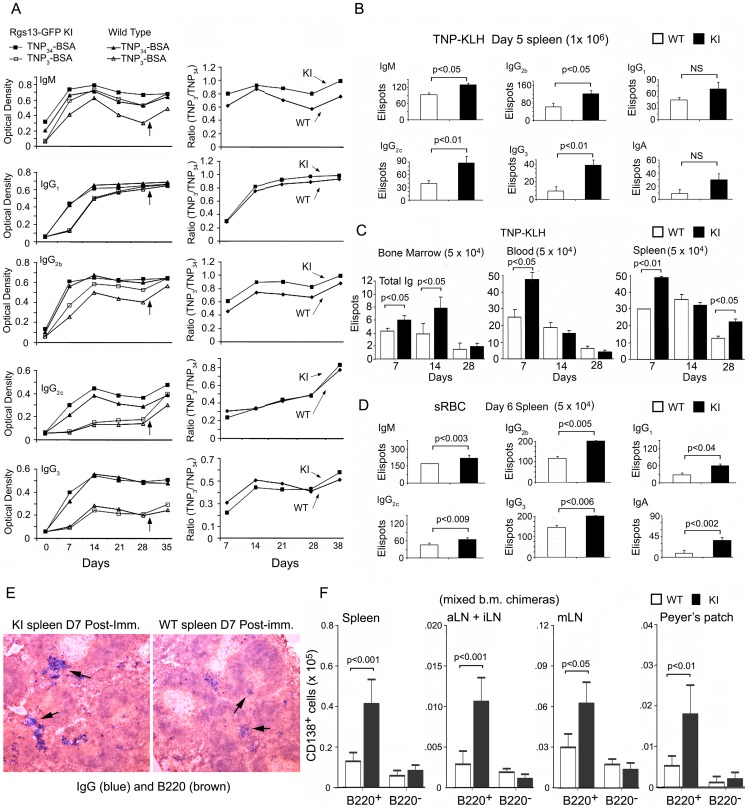
Enhanced early antibody response in the *Rgs13*GFP KI mice. A. ELISA assay results from analysis of sera collected at the indicated days from WT and KI mice immunized subcutaneously with TNP-KLH in complete Freund's adjuvant and boosted at day 28. IgM, IgG1, IgG2b, IgG2c and IgG3 specific antibodies at each time point were assayed with plates coated with TNP_34_BSA or TNP_3_BSA (left panels). The ratios between the TNP_3_ and TNP_34_ responses are shown (right panels) for the WT and KI mice. Results are from 4 WT versus 4 KI mice. Similar results were obtained from 2 additional experiments. B. ELISPOT assay results from analysis of spleen cells from 2 WT and 2 KI mice immunized 5 days previously with TNP-KLH. Similar results in 2 other experiments. Data is mean ± SEM and statistics from unpaired t tests. C. ELISPOT assay results from analysis of bone marrow, blood, and spleen cells at the indicated time points following immunization with TNP-KLH. The numbers of TNP specific ELISPOTs are shown from 1 experiment comparing 2 WT versus 2 KI mice. Similar results from one other experiment. Data is mean ± SEM and statistics from unpaired t tests. D. ELISPOT assay results from analysis of spleen cells from 2 WT and 2 KI mice immunized 6 days previously with sRBCs. Similar results in 1 other experiment. Data is mean ± SEM and statistics from unpaired t tests. E. Representative brightfield microscopy images of WT and KI spleen sections immunostained for IgG (blue) and B220 (brown). F. Flow cytometric quantification of the number of CD138^+^B220^+^ and CD138^+^B220^−^ B cells at D11 post-immunization with sRBCs in the spleens, axillary and inguinal LNs, mesenteric LNs, and Peyer's Patches of 4 mice reconstituted with a 1∶1 mix of WT and KI bone marrow 8 weeks after reconstitution. Data is mean ± SEM and statistics from unpaired t tests.

We tested the antibody responses of WT and KI mice to the thymus dependent antigen TNP-KLH. The KI mice generated a relatively normal antibody response as assessed by the induction of serum IgM, IgG, and IgA specific for TNP following immunization with TNP-KLH (Figure 7A, left panel). We noted a slight enhancement in IgM and several of the IgG isotypes at the early time points in the KI mice. To approximate the relative affinities of the antibodies from the WT and mutant mice, we measured the amount of TNP-specific antibody binding to ELISA plates coated with either TNP_3_-BSA or TNP_34_-BSA. Higher affinity antibodies will bind better to TNP_3-_BSA than will lower affinity antibodies. We found no significant difference between the serum antibodies present in serum of the KI and WT mice (Figure 7A, right panel). Since the flow cytometry results had revealed an expansion of early antibody producing cells in the KI mice we measured the number of B cells secreting specific antibodies at day 5 after immunization with TNP-KLH. Consistent with the flow cytometry data we found a modest increase in the number ELISPOTs (IgM, IgG, and IgA) using splenocytes from KI mice (Figure 7B). We also examined the number of TNP specific ELISPOTs at various days post-immunization in the bone marrow, spleen, and blood. Again we noted an increased number of TNP specific Ig secreting cells at the early time points in the bone marrow, blood, and spleen (Figure 7C). Finally, since we had done many of the experiments analyzing GC responses following the injection of sRBCs, we enumerated the number of IgM, IgG, and IgA ELISPOTs in splenocytes 6 days following intraperitoneal injection of sRBCs. As before, the KI mice generated more antibody secreting cells compared to the WT mice (Figure 7D). An increase in IgG secreting B cells is also evident from an immunohistochemical analysis of the KI versus the WT spleen 7 days post sRBC immunization (Figure 7E). Finally, we determined the absolute numbers of B220^+^CD138^+^ and B220^−^CD138^+^ cells in the spleens, peripheral LN, mesenteric LN, and Peyer's patches of the 1∶1 mixed chimeras 11 days following sRBC immunization. We found an increase in the number of B220^+^CD138^+^ B cells derived from the KI bone marrow, however, the numbers of B220^−^CD138^+^ from WT and KI bone marrow did not differ at this time point (Figure 7F).

### Cell-cell interactions likely drive the expression of *Rgs13* and its absence perturbs the normal GC B cell gene expression program

**Figure 8:**
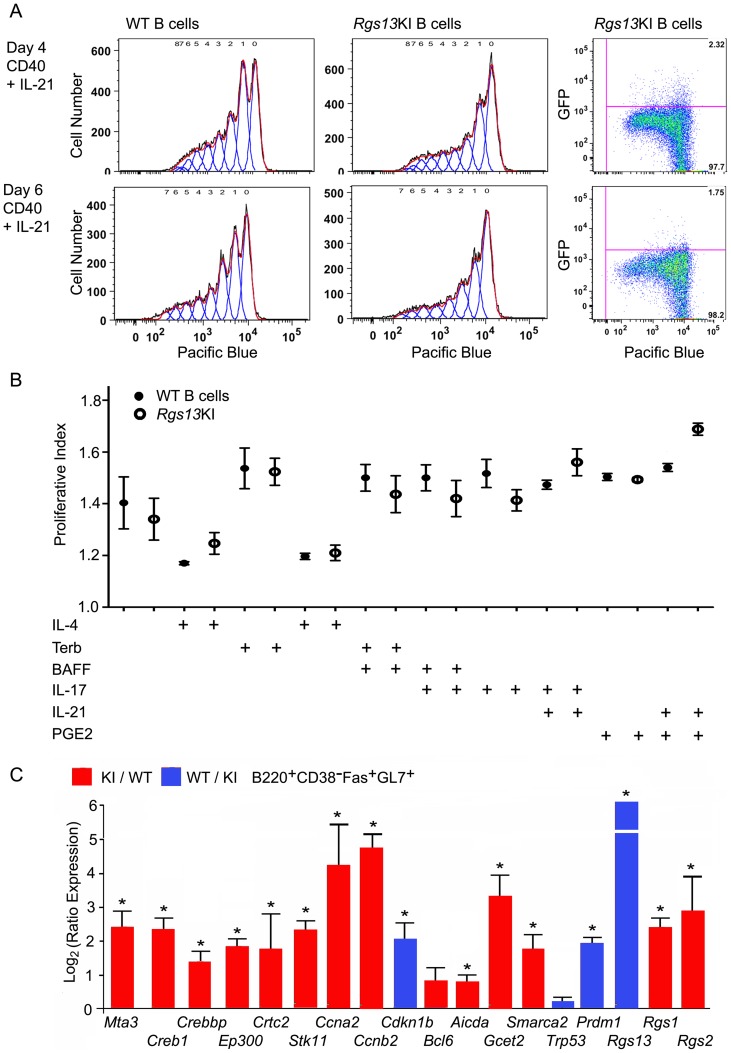
Normal B proliferation *in vitro*, but an abnormal gene expression pattern in *Rgs13*GFP KI GC B cells. A. Flow cytometric analysis of WT and KI splenic B cell proliferation following stimulation with CD40 and IL-21 for 4 or 6 days. Proliferation assessed by Pacific Blue dye dilution. Representative results from the analysis of B cells from 3 WT and 3 KI mice. An analysis of GFP versus Pacific blue for the KI B cell is shown in the far left panels. B. Proliferative indexes from the flow cytometric analysis of WT and KI mouse spleen B cells stimulated as indicated. Results mean ± SEM of 3 WT versus 3 KI B cell preparations. Similar results from 2 other experiments using a partial overlapping set of inductive signals. C. Quantitative RT-PCR using RNA extracted from spleen cells from 10 day sRBC immunized KI/WT mixed chimera mice sorted for B220^+^CD38^−^GL7^+^CD95^+^ and separated on the basis of CD45.1 or CD45.2. Results were normalized to *Gapdh* expression and expressed as ratio between KI and WT samples. Data are the mean ± SEM of triplicate values for each gene analyzed using data pooled from 3–4 separate experiments.

We expected that the GFP reporter in the *Rgs13* locus would provide an easy means to identify *in vitro* the signals that induce *Rgs13* expression *in vivo*. However, none of the inductive signals we tested *in vitro* recapitulated the high level of expression achieved *in vivo*. This included TLR ligands, anti-IgM, CD40 ligand, cytokines, chemokines, and various combinations. At best, 7% of the *in vitro* activated B cells expressed modest levels of GFP and only a rare cell achieved the level noted in the GC B cells (data not shown). Nevertheless, we tested whether we could discern a difference in the *in vitro* proliferative potential of the WT and KI B cells using a panel of different proliferative signals. Dye loaded WT and KI B cells were cultured with different inductive signals and the amount of dye dilution monitored 4 and 6 days later. A representative example of KI and WT B cells stimulated with CD40 and IL-21 is shown (Figure 8A). Analysis of GFP expression as a function of dye dilution revealed that the proliferating KI B cells maintained a slightly higher GFP expression level than did the cells that failed to divide, although as indicated above none of the cells attained the levels of GFP noted in GC B cells (Figure 8A, right panels). Perhaps because of this we found little difference in the *in vitro* proliferative potential of WT and KI B cells to a diverse set of signals (Figure 8B, data not shown).

The discrepancy between the *in vitro* and *in vivo* results led us to directly compare the WT and KI GC B cells using the 1∶1 mixed bone marrow chimeric mice. This permitted a direct comparison between the genetically distinct GC B cells in the same WT environment. We sorted B220^+^CD38^−^GL7^+^CD95^+^ B cells from the chimeric mice (CD45.1 versus CD45.2), extracted RNA, and compared gene expression by quantitative RT-PCR. The results are shown as a ratio normalized to *Gapdh* expression. Relative to WT GC B cells the KI GC B cells expressed significantly higher levels of several GC specific and cell cycle related genes and less of *Prdm1* and the cell cycle inhibitor C*dkn1b* (Figure 8C). *Rgs1* and *Rgs2* were also upregulated in the KI GC B cells. Because of the known role of CREB/CRTC2 target genes in GC B proliferation and of RGS13 in CREB mediated transcription [20], [21], we examined the expression of a number of CREB target genes as well as CREB and CREB co-activators. The KI GC B cells expressed significantly higher levels of *CREB1*, *Crebbp*, *Crtc2, Ep300*, *Stk11*, *Smarca2,* and *Mta3* (Figure 8B). *Mta3* is a Creb/Crtc2 target gene that encodes a protein that physically interacts with BCL6 and appears to be instrumental in maintaining the GC B cell transcriptional program that precludes premature plasma cell differentiation [32]. These results indicate that the loss of *Rgs13* impacts a genetic program that is known to controls GC B cell proliferation, self-renewal, and differentiation [21].

## Discussion

Many RGS proteins are broadly expressed potentially impacting GPCR signaling in many cell types. In contrast *Rgs13* exhibits a restricted expression pattern predominately within a limited number of cell types in the hematopoietic system including mast cells and GC B cells [16], [18]. It has also been reported in follicular helper T cells in humans [28] although we found no evidence of GFP expression in mouse follicular helper T cells. Using GFP as a surrogate marker for *Rgs13* expression we documented expression in recently activated B cells, the majority of GC dark and light zone cells, many early switched B cells, but not in long term switched B cells or plasma cells. GFP expression correlated with the expression of both cell cycle and GC specific genes. The loss of *Rgs13* led to an expansion of the GC compartment at sites of constitutive immune activation and following exogenous antigen administration, an enhanced early plasma cell response, and the production of large GCs. A comparison of gene expression between WT and KI GC B cells led to the discovery that the loss of *Rgs13* distorted the normal gene regulatory program that is controlled by CREB and the CREB co-activator CRTC2 [21].

While expression of GFP in the KI mouse GC B cells mirrored that expected based on *Rgs13* RT-PCR and previous microarray studies the rapid induction of GFP in several percent of the KI B cells following immunization was a surprise. At one day post sRBC immunization nearly 4% of the splenic and 2% of the LN B cells had acquired GFP expression. Studies with transgenic B cells have indicated that within hours of antigen engagement B cells upregulate CCR7 and begin to shift to the B/T border and to interfollicular zones [2]. The rapid induction of GFP *in vivo* suggested that Ig receptor signaling might lead to *Rgs13* expression. Perplexingly none of the signals or combination of signals we tried *in vitro* recapitulated the GFP expression we had observed with freshly isolated GC B cells. Further studies are on-going to determine the specific signals that trigger *Rgs13* expression. A direct T-B cell interaction may be required to trigger *Rgs13* expression. A human microarray study has also indicated that *RGS13* is induced prior to cells acquiring a full GC phenotype [33]. Supporting our flow cytometry results we noted a rapid induction of GFP expression by multiphoton intravital microscopy in cells located at the T-B border of inguinal LNs 1 day post immunization. Even at this early time point clusters of GFP positive cells could be observed. Arguing that antigen receptor signaling is insufficient to trigger GFP expression, immunization of the KI mice with the thymus independent antigen TNP-Ficoll did not induce GFP positive B cells *in vivo* (C. P., unpublished observation). It appears that the induction of GFP in the *Rgs13*GFP KI B cells serves as an early marker of B cells that have interacted with T cells.

Since *Rgs13* is not induced by antigen receptor signaling it may not impact the initial EBI2 and CCR7 dependent localization of activated B cells to the T-B border and interfollicular sites. At these sites the activated B cells form long lasting conjugates with cognate CD4 T cells, which results in extensive B cell proliferation and shape subsequent B cell fate decisions. Imaging the KI mice after immunization revealed an expanding knot of GFP positive cells that expanded over the first 3 days post-immunization. In WT mice RGS13 expression in these activated and proliferating B cells should lessen Gα_i_ signaling. While G-protein signaling affects cell fate decisions in the central nervous system and contributes to asymmetric cell divisions in model organisms [34], [35], we did not see any obvious bias in initial cell fate destiny, but rather an overall expansion of the number of cells destined to become extra-follicular plasma cells, GC independent memory cells, and GC B cell precursors. Apparently, *Rgs13* expression normally limits the expansion of activated B cells at the T-B cell border. In its absence precursors expand more rapidly generating additional cells that eventually enter into these different differentiation pathways.

Both a GC dependent and GC independent pathway can generate memory B cells following immunization. The GC independent pathway rapidly generates IgM and switched memory cells, which consistent with their non-GC origin have relatively un-mutated antigen receptors. These cells arise from activated, naive B precursors located at the T-B border that co-express CD38 and GL7 [25]. While we noted increased numbers of B220^+^CD38^+^GL7^+^ cells at the D3/4 post immunization in the KI mice at D2 only 15% of these cells co-expressed GFP and at D5 40%. Rather at the early time points post-immunization many of the GFP^+^ cells remained in the B220^+^CD38^+^GL7^−^ gate. These early GFP positive CD38^+^GL7^+^ and CD38^+^GL7^−^ B cells expressed an intermediate level of GFP, less than the typical germinal center B cell found later in the immunized KI mice. At D4/5 post immunization we noted a significant expansion in the numbers of B220^+^CD38^+^IgG1^+^ cells in both the WT and KI mice although 2–3 fold more in the KI mouse. Yet again only about 20% of these cells were GFP^+^. Whether some of these cells had briefly expressed GFP or never expressed GFP needs to be resolved. We are developing a new mouse strain where the *Rgs13* coding region has been replaced with the Cre recombinase to address this question as well as others. Long term switched memory B cells expressed GFP at levels similar to those present in naïve B cells.

B220^+^CD38^+^GL7^+^ cells also serve as precursors for GC B cells as they return from the interfollicular region to the center of the LN follicle or splenic B cell area to establish a nascent GC. Our intravital microscopy identified KI GFP positive cells moving from the interfollicular zone into the LN follicle and then expanding within the follicle. By day 11 post immunization most of the KI GC B cells and CD38^−^ IgG1 switched B cells expressed GFP. Although nearly 90% of centrocytes and centroblasts expressed high levels of GFP 10% of both populations lacked GFP expression. Since dark zone and light zone B cells largely remain confined to their respective regions and only occasionally move between zones, a decrease in RGS13 expression might be predicted to augment responsiveness to a CXCL12 or CXCL13 gradient facilitating cycling between the zones. In the KI mice the lack of RGS13 might perturb the cycling of cells between zones as both centroblasts and centrocytes would remain sensitive to chemokines. This is predicted to alter GC zoning [10], a result consistent with the morphological abnormalities noted in the spleens and LNs of the immunized KI mice. However, we could find little *in vitro* evidence to support a heightened sensitivity of the *Rgs13* deficient GC B cells to chemokines. This failure may be secondary to intrinsic problems in the *in vitro* chemotaxis assays as well as the tendency of GC B cells to undergo apoptosis *ex vivo*. We did note that the KI GC B cells that lacked GFP expression had a heightened response to chemokines compared to those that expressed GFP. As GC B cells express other RGS proteins there could be some compensation for the loss of RGS13. In fact the expression of *Rgs1* and *Rgs2*, both located close to the *Rgs13* locus on chromosome 1, were increased in GC B cells from the KI mice. It will be of interest to examine the GC zoning in mice with a *Gnai2* G184S KI, which interferes with the binding of all RGS proteins to Gα_i2_ [36].

Besides the early increase in GC B cell precursors in the KI mice, the numbers of splenic GC B cells increased more rapidly than they did in the WT mice. Furthermore the KI mice had more GC B cells than did WT mice in their Peyer's patches and mesenteric lymph nodes, sites of ongoing immune reactivity to commensal bacterial products. The more robust GC response in the spleen to an exogenous antigen the ongoing exaggerated GC response at mucosal sites seemed less likely to be explained by heightened heterotrimeric G-protein signaling and more likely as a consequence of RGS13's role in the cell nucleus to limit CREB signaling. To investigate the exaggerated GC response in the KI mice, we initially sorted B220^+^GFP^+^ and B220^+^GFP^−^ cells from KI mice D8-10 following immunization, and from B220^+^ B cells from unimmunized WT mice, we extracted RNA, and performed microarrays. The genes known to be upregulated in GC B cells all appeared in the KI GFP positive cells. In addition, the cell cycle specific genes were upregulated only in the B220^+^GFP^+^ cells while the gene expression profile of the B220^+^GFP^−^ KI B cells from immunized mice resembled the WT B220^+^ cells from unimmunized mice. Thus, GFP expression in the KI animals marked nearly all the proliferating B cells at D8-10 days post immunization (unpublished observations). Because of difficulties in comparing GC B cells from different WT and KI animals, we created bone marrow chimeric mice and sorted the GC B cells following immunization. Based on the quantitative PCR analysis of RNA extracted from the genetically distinct cells we found lower levels of *Prdm1*, which promotes B cell differentiation, and *Cdkn1b*, a known cell cycle inhibitor in the KI GC B cells as compared to WT GC B cells. In addition, we found higher levels of the several GC specific genes, cell cycle specific genes, and a number of CREB related and CREB target genes.

A subset of CREB target genes have emerged as important regulators of B cells within human GCs [21]. In GC B cells exogenous and intrinsic AID-induced DNA strand breaks activate a signaling pathway that inactivates CRTC2, a transcriptional co-activator of CREB. CRTC2 inactivation represses a genetic program that controls GC B cell proliferation and differentiation. CRTC2 translocation to the nucleus increases GC B cell proliferation and reduces antibody production while CRTC2 inactivation promotes B cell differentiation [21]. Ca^2+^ and cAMP signaling can promote RGS13 accumulation in the nucleus, where it forms a complex with phosphorylated CREB and CBP/p300, which suppresses CREB-mediated gene expression [20]. In the absence of RGS13, CREB and its co-activators will likely increase the transcription of downstream target genes. *Mta3*, *Aicda*, and *Smarca4* are target genes of CREB/CRTC2 in human GC B cells [21] and more highly expressed in the KI GC B cells than in WT GC B cells. Interestingly, MTA3 is a cell-type specific component of the Mi-2-NURD transcriptional co-repressor complex that is expressed GC B cells. MTA3 physically interacts with BCL6 and helps maintain the GC B cell transcriptional program that promotes GC B cell proliferation and limits B cell differentiation into plasma cells [32]. Thus, the loss of RGS13 likely facilitates ongoing GC B cell proliferation explaining the large GCs and increased number of GC B cells found in the KI mice.

Together these data indicate that RGS13 has several roles in B cells. It limits the initial expansion of recently activated B cells and the expansion of GC B cells. It likely does so in part by altering CREB signaling. In addition, it along with other RGS proteins help to coordinate the responsiveness of recently activated and GC B cells to chemoattractants. The *Rgs13*GFP KI mice along with other engineered mice should allow a better understanding of how RGS13 and other RGS proteins accomplish this. While we did not note any frank autoimmunity or increased incidence of lymphoma in the KI mice crossing these mice onto different genetic backgrounds with a predilection towards autoimmunity or lymphomagenesis should be of interest.

## Supporting Information

Figure S1 **Construction of the** ***Rgs13*** **GFP KI mouse and the loss of** ***Rgs13*** **expression.** A. Schematics of Rgs13 genomic, targeted, and Cre deleted loci. B. Southern blot demonstrating the Rgs13 targeting and Cre mediated deletion of the Neomycin gene. C. Standard RT-PCR examining the expression of *Rgs1, Rgs10*, *Rgs13*, and *Rgs19* in immunized splenic B cells or Peyer's Patch B cells from WT and KI mice.
(TIF)

Figure S2 **Initial assessment of cell populations in** ***Rgs13*** **GFP KI mice.** A. Flow cytometric analysis of B cell development in WT and KI bone marrow. The percentage of GFP expression in the various subsets in the KI cells is indicated. The analysis was performed on bone marrow from 4 WT versus 4 KI animals. Data is mean ± SEM and statistics from unpaired t tests. B. Coulter counter and blood smear analysis of blood from 10 WT and 10 KI animals. C. Flow cytometric analysis of B cell subsets in the spleen of WT and KI mice. The percentage of GFP expression in the various subsets in the KI cells is indicated. The analysis was performed on spleens from 4 WT versus 4 KI animals. Data is mean ± SEM and statistics from unpaired t tests. D. Flow cytometric analysis of B1 and B2 B cells in the peritoneum of WT and KI mice. The percentage of GFP expression in the various subsets in the KI cells is indicated. The analysis was performed on peritoneal cells from 4 WT versus 4 KI animals. Data is mean ± SEM and statistics from unpaired t tests.
(TIF)

Figure S3 **Follicular helper T cells do not express high levels of GFP in the** ***Rgs13*** **GFP KI mice.** A. Flow cytometric analysis of the number of follicular helper cells in the spleen and Peyer's patches of WT and KI animals. The % of follicular helper T cells (CD4^+^B220^−^PD-1^+^CXCR5^+^) in the CD4 gate from the analysis of cells from the spleens and Peyer's patches of sRBC immunized animals either 11 or 30 days post immunization. Analysis is from 4 WT versus 4 KI animals. Data is mean ± SEM and statistics from unpaired t tests. B. Representative flow cytometry plots examining the expression of GFP in follicular helper T cells from the D11 immunized spleen, mesenteric LN, and Peyer's patches. C. Flow cytometric analysis of the number of follicular helper T cells in mixed bone marrow chimeras derived from either WT or KI bone marrow. Cells obtained from 4 chimeric mice at 11 days post sRBC immunization. Data is mean ± SEM. No statistical difference was noted.
(TIF)

Video S1 **Intravital microscopy of the inguinal lymph node from a** ***Rgs13*** **GFP KI mouse 1D post-immunization identifies GFP positive cells located at the edge of B cell follicle.** Shown is the distribution and behavior of GFP expressing KI cells in the inguinal lymph node of a KI mouse. Images were taken at D1 post immunization via an intra-peritoneal injection of sRBCs. An image sequence of an 80 μm z- projection was acquired with 20× lens over 30 minutes. From shallow (top left) to deep (low right), each panel is from a 80 μm volume image stack at 10 μm intervals moving from the LN surface deeper into the lymph node cortex. The capsule of the lymph nodes was visualized by second harmonic signal (blue) from collagen. Adoptively transferred B cells labeled with CMTMR are shown in red. The GFP signals are shown in the green channel. White lines indicate edge of the B-cell follicle, which was based on the distribution of adoptively transferred WT B-cells. The scale bar is 200 μm. Time counter is h:min:sec.
(MOV)

Video S2 **Intravital microscopy of the inguinal lymph node from a** ***Rgs13*** **GFP KI mouse 2D post immunization identifies an expanding population of GFP positive cells located at the edge of B cell follicle.** Shown is the distribution and behavior of GFP expressing KI cells in the inguinal lymph node of a KI mouse. Images were taken at D2 post immunization via intra-peritoneal injection of sRBCs. An image sequence of an 80 μm z- projection was acquired with 20× lens over 30 minutes. From shallow (top left) to deep (low right), each panel is from a 80 μm volume image stack at 10 μm intervals moving from the LN surface deeper into the lymph node cortex. The capsule of the lymph nodes was visualized by second harmonic signal (blue) from collagen. Adoptively transferred B cells labeled with eFluor® 670 are shown in white. The follicular dendritic cell (FDC) network (red) was visualized by means of subcutaneous injection of anti-CD21/35 PE conjugated antibody (3 μg) prior to imaging. GFP signals are shown in the green channel. White lines indicate edge of the B-cell follicle based on distribution of WT B cells adoptively transferred 1D prior to imaging. The scale bar is 150 μm. Time counter is h:min:sec.
(MOV)

Video S3 **Intravital microscopy of the inguinal lymph node from a** ***Rgs13*** **GFP KI mouse 8D post immunization identifies a large population of GFP positive cells residing in a germinal center located in the LN follicle.** Shown is the distribution and behavior of GFP expressing KI cells in the LN follicle of the inguinal lymph node of a KI mouse. Images were taken D8 post immunization via intra-peritoneal injection of sRBCs. An image sequence of an 80 μm z- projection was acquired with 25x lens over 30 minutes. From shallow (top left) to deep (low right), each panel is from the 80 μm volume image stack at 10 μm intervals moving from the LN surface deeper into the lymph node cortex. The capsule of the lymph nodes was visualized by second harmonic signal (blue) from collagen. Adoptively transferred B cells labeled with CMF_2_HC are shown in blue. The follicular dendritic cell (FDC) network (red) was visualized by means of subcutaneous injection of anti-CD21/35 PE conjugated antibody (3 μg) prior to imaging. GFP signals are in the green channel. White lines indicate edge of the B-cell follicle, and were drawn based on distribution of WT B cells adoptively transferred 1D prior to imaging. The scale bar is 200 μm. Time counter is h:min:sec.
(MOV)
