# Supplementary figures and images for: Rgs13 Constrains Early B Cell Responses and Limits Germinal Center Sizes
Source: PLoS One. 2013 Mar 22;8(3):e60139. doi: 10.1371/journal.pone.0060139 (PMC3606317; doi:10.1371/journal.pone.0060139)

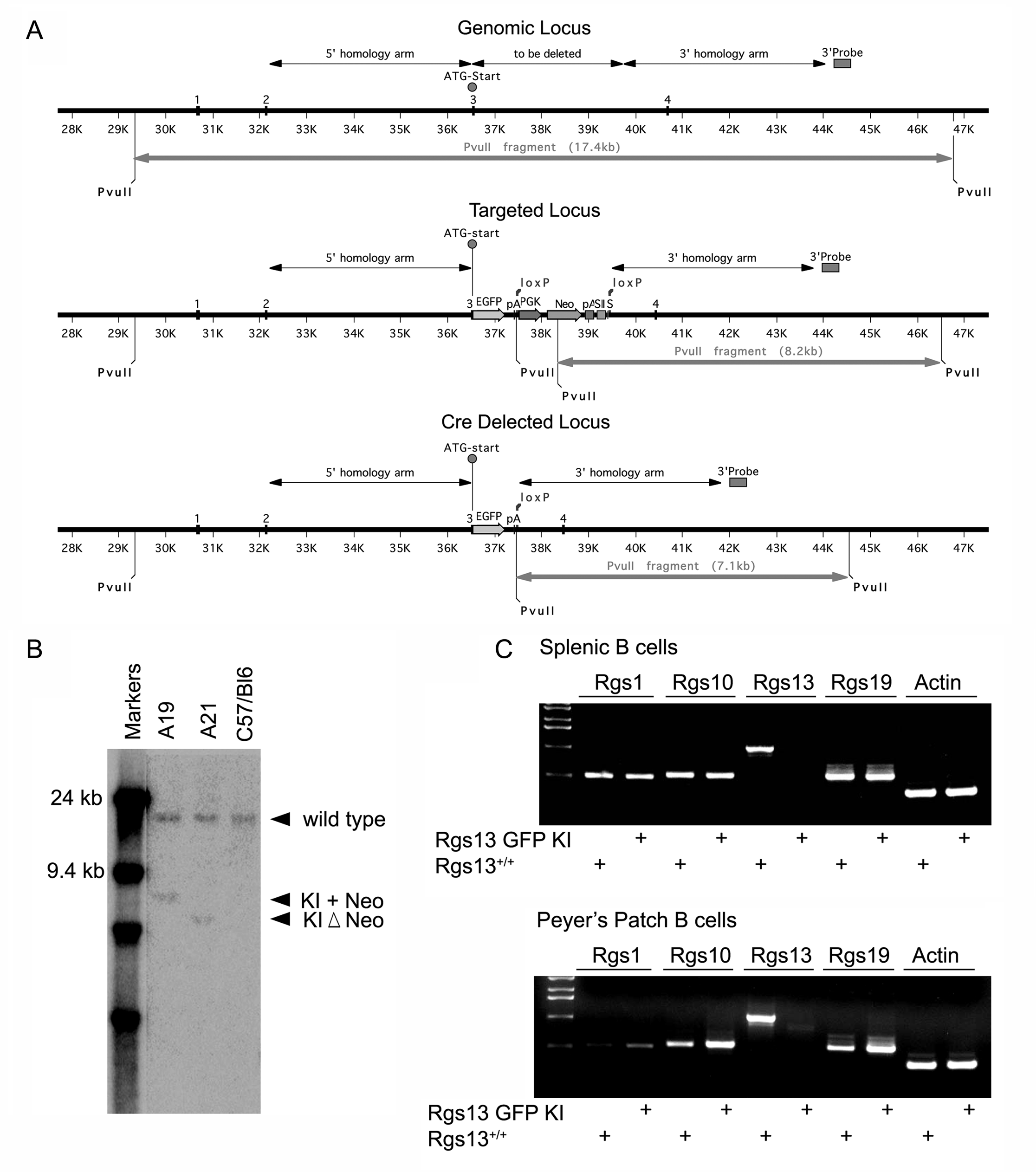

Supplement: Figure S1 — Construction of the Rgs13 GFP KI mouse and the loss of Rgs13 expression. A. Schematics of Rgs13 genomic, targeted, and Cre deleted loci. B. Southern blot demonstrating the Rgs13 targeting and Cre mediated deletion of the Neomycin gene. C. Standard RT-PCR examining the expression of Rgs1, Rgs10, Rgs13, and Rgs19 in immunized splenic B cells or Peyer's Patch B cells from WT and KI mice. (TIF) [file pone.0060139.s001.tif]

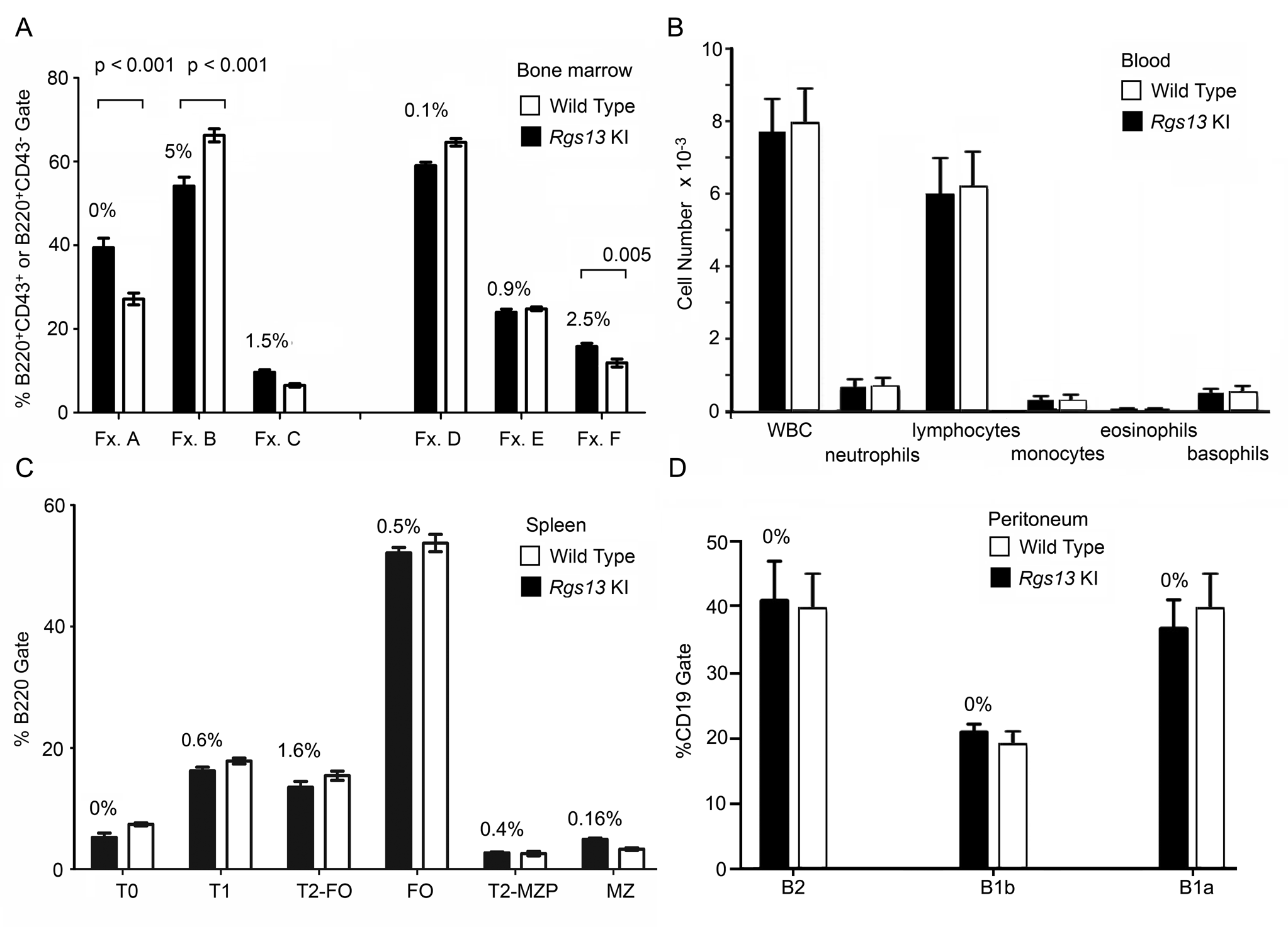

Supplement: Figure S2 — Initial assessment of cell populations in Rgs13 GFP KI mice. A. Flow cytometric analysis of B cell development in WT and KI bone marrow. The percentage of GFP expression in the various subsets in the KI cells is indicated. The analysis was performed on bone marrow from 4 WT versus 4 KI animals. Data is mean ± SEM and statistics from unpaired t tests. B. Coulter counter and blood smear analysis of blood from 10 WT and 10 KI animals. C. Flow cytometric analysis of B cell subsets in the spleen of WT and KI mice. The percentage of GFP expression in the various subsets in the KI cells is indicated. The analysis was performed on spleens from 4 WT versus 4 KI animals. Data is mean ± SEM and statistics from unpaired t tests. D. Flow cytometric analysis of B1 and B2 B cells in the peritoneum of WT and KI mice. The percentage of GFP expression in the various subsets in the KI cells is indicated. The analysis was performed on peritoneal cells from 4 WT versus 4 KI animals. Data is mean ± SEM and statistics from unpaired t tests. (TIF) [file pone.0060139.s002.tif]

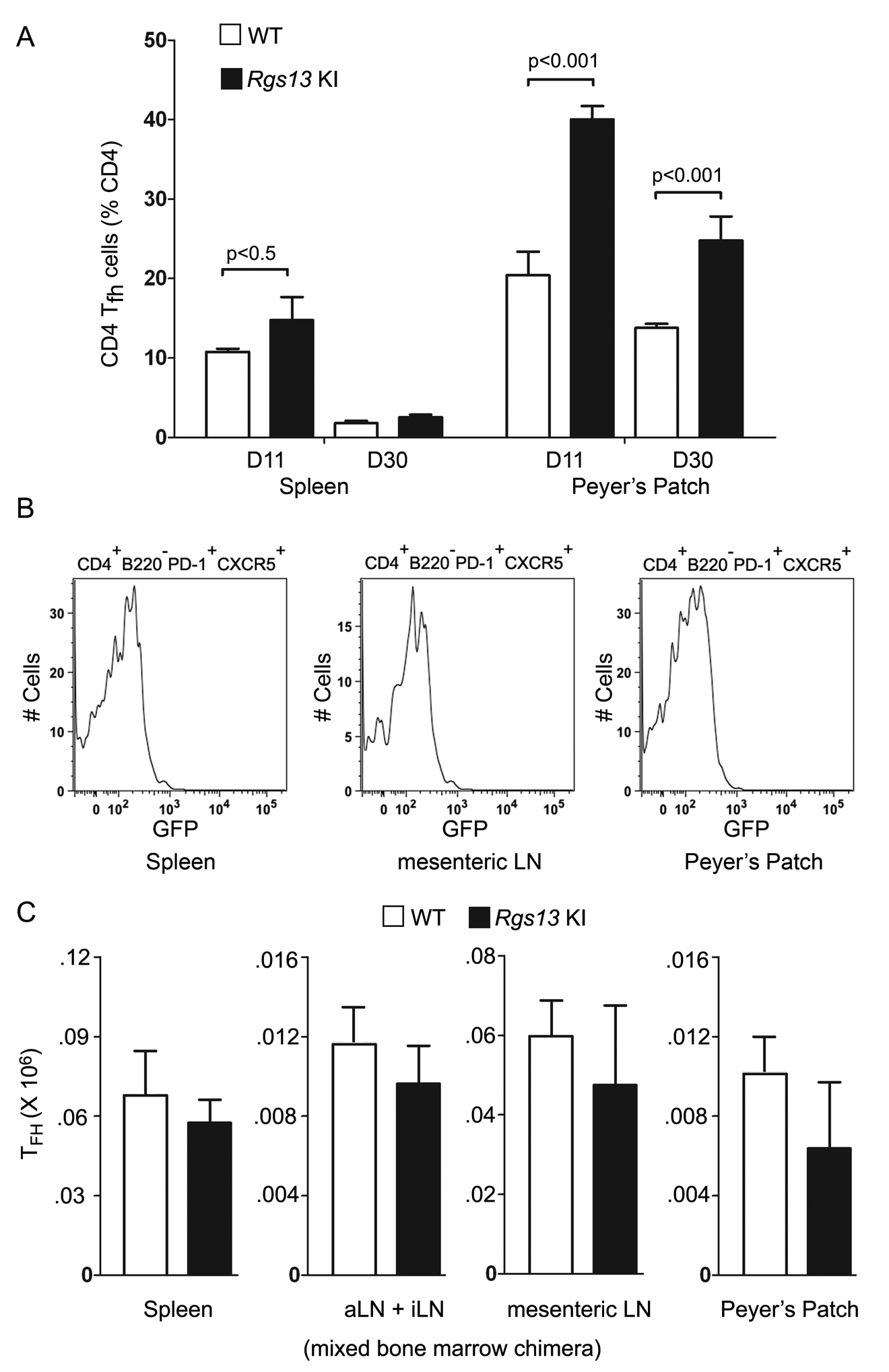

Supplement: Figure S3 — Follicular helper T cells do not express high levels of GFP in the Rgs13 GFP KI mice. A. Flow cytometric analysis of the number of follicular helper cells in the spleen and Peyer's patches of WT and KI animals. The % of follicular helper T cells (CD4+B220−PD-1+CXCR5+) in the CD4 gate from the analysis of cells from the spleens and Peyer's patches of sRBC immunized animals either 11 or 30 days post immunization. Analysis is from 4 WT versus 4 KI animals. Data is mean ± SEM and statistics from unpaired t tests. B. Representative flow cytometry plots examining the expression of GFP in follicular helper T cells from the D11 immunized spleen, mesenteric LN, and Peyer's patches. C. Flow cytometric analysis of the number of follicular helper T cells in mixed bone marrow chimeras derived from either WT or KI bone marrow. Cells obtained from 4 chimeric mice at 11 days post sRBC immunization. Data is mean ± SEM. No statistical difference was noted. (TIF) [file pone.0060139.s003.tif]
